# A prospective study of associations between early fearfulness and perceptual sensitivity and later restricted and repetitive behaviours in infants with typical and elevated likelihood of autism

**DOI:** 10.1177/13623613211068932

**Published:** 2022-01-13

**Authors:** Nisha Narvekar, Virginia Carter Leno, Greg Pasco, Mark H Johnson, Emily JH Jones, Tony Charman

**Affiliations:** 1 King’s College London, UK; 2 Birkbeck, University of London, UK; 3 University of Cambridge, UK

**Keywords:** autism, early development pathways, elevated likelihood, restricted and repetitive behaviours, temperament

## Abstract

Autism is diagnosed based on social and communication difficulties, restricted and repetitive behaviours and sensory anomalies. Existing evidence indicates that anxiety and atypical sensory features are associated with restricted and repetitive behaviours, but cannot clarify the order of emergence of these traits. This study uses data from a prospective longitudinal study of infants with and without a family history of autism (*N* = 247; Elevated Likelihood *N* = 170 and Typical Likelihood *N* = 77). Longitudinal cross-lag models tested bidirectional pathways between parent-rated infant fear/shyness and perceptual sensitivity at 8, 14 and 24 months, and associations between these domains and parent-rated restricted and repetitive behaviours and social communication scores at 36 months. In addition to within-domain continuity, higher levels of fear/shyness at 14 months were associated with higher levels of perceptual sensitivity at 24 months. Higher levels of both fear/shyness and perceptual sensitivity at 24 months were associated with greater restricted and repetitive behaviours and social communication scores at 36 months. Results demonstrate the directionality of developmental pathways between fear/shyness and perceptual sensitivity in infancy and toddlerhood, but question theories that argue that these domains specifically underlie restricted and repetitive behaviours rather than autism. Identifying how early emerging anxiety and sensory behaviours relate to later autism is important for understanding pathways and developing targeted support for autistic children.

**Lay abstract:**

Restricted interests and repetitive behaviours are central to the diagnosis of autism and can have profound effects on daily activities and quality of life. These challenges are also linked to other co-occurring conditions such as anxiety and sensory sensitivities. Here, we looked at whether early emerging signs of anxiety and sensory problems appear before symptoms of autism by studying infants with a family history of autism, as these infants are more likely to develop autism themselves. Studying infant siblings provides an opportunity for researchers to focus on early developmental markers of autism as these infants can be followed from birth. This study found that early infant signs of anxiety (e.g. fear/shyness) predicted later perceptual sensitivity, and those infants who scored higher on fear/shyness and sensitivity were more likely to experience more persistent repetitive behaviours, but also social and communication difficulties in toddlerhood. Early signs of anxiety and perceptual sensitivity may thus relate to both later social difficulties and repetitive behaviours. These findings support the importance of further research exploring the causal links between these domains in relation to autism, resulting in increased understanding of children who go onto develop autism in the future and guiding early interventions and supports.

Autism spectrum disorder (ASD) (henceforth referred to as autism) is a neurodevelopmental condition with a childhood prevalence of ~1.5% (Maenner et al., 2020) which is typically diagnosed approximately around 6 years of age (Brett et al., 2016). The core characteristics involve a varying degree of difficulty in social functioning, communication, and the presence of restricted and repetitive behaviours and sensory anomalies (American Psychiatric Association [APA], 2013). Although restrictive and repetitive behaviours (RRBs) are part of the diagnostic criteria for autism, historically more attention has been given to social and communication difficulties, and therefore, less is known about RRB despite their influence on daily activities (Leekam et al., 2011) and quality of life (Hochhauser & Engel-Yeger, 2010). RRBs are a mix of behaviours characterised by repetition and desire for sameness in the environment, preoccupation with parts of objects, restricted interests and ritualistic behaviours, and the most recent diagnostic criteria now includes hyper- or hyporeactivity to sensory input or unusual interests in sensory aspects of the environment within the RRB domain (APA, 2013). RRBs are present at an early age (Wolff et al., 2014), including in typically developing infants (Leekam et al., 2007), and yet remain understudied compared to early social motivation and social attention (Elsabbagh & Johnson, 2016). A more comprehensive understanding of the infant characteristics associated with individual differences in RRB could identify individuals who may have more difficulty in this domain and inform the development of better targeted support.

## The association between anxiety and RRB in autistic individuals

Recent research has reported associations between anxiety (which is highly prevalent in autistic youth; Simonoff et al., 2008) and RRB in autistic children (Gotham et al., 2013; Lidstone et al., 2014; Rodgers et al., 2012). Studies of typically developing children with the anxiety-related condition obsessive–compulsive disorder (OCD) also suggest a link between anxiety and repetitive behaviours that may extend beyond autism. Indeed, children with OCD and autistic children are found to have comparable levels of sameness behaviours such as ritualistic habits and adherence to routines, and repetitive movements (Jiujias et al., 2017; Zandt et al., 2007), although the drivers of RRB may differ between the two groups. One interpretation of these findings is that certain types of RRB could be useful for managing anxiety levels by allowing the child to impose control over their environment (Lidstone et al., 2014; Rodgers et al., 2012). Such frameworks assume that anxiety precedes RRB. However, a few studies have tested the directionality of the anxiety–RRB association; two studies that assessed RRB early in childhood found higher RRB was associated with greater anxiety later in development in autistic individuals (Baribeau et al., 2020; Ben-Itzchak et al., 2020), but the anxiety to RRB pathway was not tested.

An important factor to consider when examining associations between anxiety and RRB is sensory processing differences, broadly characterised as hypo- and hypersensitivity to sensory input and/or sensation-seeking, although others note that measurements of sensory sensitivity often conflate sensory sensitivity (i.e. differences in ability to detect differences in sensory input) and sensory reactivity (i.e. observable reactions to sensory input, which may be in part due to greater affective response) (Schulz & Stevenson, 2020). As noted above, while sensory processing atypicalities are now included as a subdomain of RRB symptoms in autism (APA, 2013), some suggest that sensory processing differences may themselves directly contribute to individual differences in other aspects of the RRB domain (Boyd et al., 2010; Grzadzinski et al., 2020; Schulz & Stevenson, 2019). Sensory sensitivities are also positively associated with anxiety in autistic and typically developing children (Neil et al., 2016), and sensory over-responsivity (akin to hyper-sensitivity) is associated with longitudinal changes in anxiety in autistic toddlers (Green et al., 2012). Although most research has focused on direct pathways between sensory processing and RRB, or between sensory processing and anxiety, it is also possible, if not likely, that more complex associations exist. For example, it may be that the early emerging sensory aspects of the RRB domain trigger anxiety, which in turn triggers other behavioural RRB characteristics (e.g. repetitive and stereotypic behaviours, and insistence on sameness) in order to regulate arousal. Thus, it may be that motoric/behavioural RRB is a proximal response to both anxiety and sensory sensitivities (and the two co-occur), or that sensory sensitivities precede anxiety (or vice versa), which in turn prompt RRB (Joosten et al., 2009). Indeed, studies that have measured all three constructs in autistic children report that the association between anxiety and RRB may be partially mediated by sensory avoidance sensitivity (Black et al., 2017; Lidstone et al., 2014), or that sensory hypo- and hypersensitivity act upon RRB through anxiety (Wigham et al., 2015), although all used a cross-sectional design.

## Prospective infant sibling design

Most research on the interplay between anxiety, sensory processing and RRB in autism has been conducted with individuals with an existing diagnosis and is largely cross-sectional. However, reported associations in older children are likely compounded by a history of interactions between these factors once they have emerged earlier in development (Johnson et al., 2015). Thus, focusing on these factors early in development allows one to examine associations between domains as individual differences in factors of interest emerge. Teasing apart issues of directionality is required to build a more mechanistic model of pathways to RRB. This may be possible within prospective studies of infants with a family history of autism, which enable the measurement of particular phenotypes before diagnosis occurs (Constantino et al., 2021; Jones et al., 2014). About 20% of infants with an older sibling with autism will go on to receive a diagnosis of autism themselves (Ozonoff et al., 2011), and another 20% will manifest subthreshold symptoms or developmental delay (Charman et al., 2017; Messinger et al., 2013). This recurrence rate allows the feasible study of emerging autism in siblings with (referred to as Elevated Likelihood; EL) and without (referred to as Typical Likelihood; TL) a first-degree relative with an autism diagnosis followed from the first year of postnatal life to an age at which a diagnosis can be made. Although only a subgroup of these infants will go onto receive a diagnosis, on a whole these cohorts are characterised by substantial variation in autistic traits. Given that genetic studies find that aetiological influences on autism traits at the extremes are shared with aetiological underpinnings of traits in general population (Robinson et al., 2011) (i.e. that aetiology of the diagnosis is shared with aetiology of traits), studying precursors of continuous autistic traits can identify mechanisms relevant to autism as a diagnostic category.

When studying anxiety and sensory processing differences, it is necessary to identify appropriate developmental precursors to later clinically defined responses (Clifford et al., 2013; Macari et al., 2017; Schwichtenberg et al., 2013). In typically developing children, research suggests temperamental styles such as behavioural inhibition, fearful reactivity to novel stimuli and shyness precede childhood anxiety disorders (Möller et al., 2016). Although less is known about normative developmental patterns of sensory processing and manifestations of atypical perceptual processing in early infancy, perceptual sensitivity has been conceptualised as a core component of models of infant temperament and appears to be stable between infancy and toddlerhood (Gartstein & Rothbart, 2003; Putnam et al., 2006).

## Present study

This study uses a prospective longitudinal design to examine associations between fear/shyness, perceptual sensitivity, RRB and social communication in EL and TL infant siblings in early infancy. This study has two aims: first, to test bidirectional associations between fear/shyness and sensory processing between 8 and 24 months using well-validated measures of infant characteristics; second, to test if these constructs are associated with RRB and social communication at 36 months of age. We include social communication as an outcome to assess the specificity of associations with RRB; the constructs of interest (anxiety and sensory processing) are also reported to be longitudinally associated with social communication (Vlaeminck et al., 2020).

## Methods

### Participants

As part of the British Autism Study of Infant Siblings (BASIS: http://www.basisnetwork.org), 247 infants were assessed four times, with visits at approximately 6–9, 12–15, 24 and 36 months of age. Infants in the EL group (*n* = 170; 85 male; 85 female) had at least one older sibling with a community clinical diagnosis of autism, which was confirmed based on parent report: using the Development and Wellbeing Assessment (DAWBA; Goodman et al., 2000), the Social Communication Questionnaire (SCQ; Berument et al., 1999) or parent confirmed community clinical autism diagnosis. Infants in the TL group (*n* = 77; 35 male and 42 female) had at least one older sibling with typical development and no known autism in first-degree family members (as confirmed through parent interviews regarding family medical history). The Mullen Scales of Early Learning (MSEL; Mullen, 1995) and Vineland Adaptive Behaviour Scale-II (VABS-II; Sparrow et al., 2005) were administered at each visit. All toddlers were assessed at 24 and 36 months with the Autism Diagnostic Observation Schedule-2 (ADOS-2; Lord et al., 2012), and at 36 months, parents were interviewed using the Autism Diagnostic Interview–Revised (ADI-R; Lord et al., 1994). Best estimate clinical diagnosis of autism was made at age 3 informed by, but not dependent on outcomes from the ADOS-2, the ADI-R, the VABS-II and MSEL scores by experienced researchers (T.C. and G.P.). Thirty-four EL infants met the diagnostic criteria for autism at 36 months (see Supplementary Table S1). Participants were recruited from a volunteer database at the Birkbeck Centre for Brain and Cognitive Development. All parents included in the study completed written informed consent before each visit.

### Measures

Fear and sensory sensitivity were assessed with the Infant Behaviour Questionnaire-Revised (IBQ-R; Gartstein & Rothbart, 2003) at 8 and 14 months and Early Childhood Behavioural Questionnaire (ECBQ; Putnam et al., 2006) at 24 months. Both measures are reliable and well-validated parent-reported questionnaires. Internal consistency and inter-rater reliability of the IBQ-R have been previously investigated by Gartstein and Rothbart (2003), where internal consistency was acceptable to excellent for all IBQ-R subscales in children aged 3–9 months (*α* = 0.70–0.90). Parents rated their child on how often they exhibited each behaviour in the previous 2 weeks. Items are scored on a Likert-type scale from 1 (Never) to 7 (Always). The IBQ-R is designed for infants aged 3–12 months and consists of 191 items. The ECBQ is developed to assess temperament-related behaviours in children aged 18–36 months and consists of 201 items. The IBQ-R subscales of fear (16 items) and perceptual sensitivity (12 items), the ECBQ subscales of shyness (12 items) and perceptual sensitivity (12 items) were calculated. The perceptual sensitivity subscale refers to detection or perceptual awareness of slight, low-intensity stimulation from the external environment. In the IBQ-R, the fear subscale measures infant distress or inhibited approach to novel social and non-social stimuli. In the ECBQ, this collection of behaviours is separated into two subscales termed fear (indexing distress or inhabited approach to novel non-social stimuli) and shyness (indexing discomfort, slow or inhibited approach to novelty and uncertainty in social situations) – development work suggested the social and non-social components could not be reliably dissociated in infancy (Gartstein and Rothbart, 2003). We chose the IBQ-R fear and ECBQ shyness subscales as the closest measures to the construct of infant/toddler behavioural inhibition that maps onto later childhood anxiety, as per previous research involving typically developing infants (Dyson et al., 2011) and in EL samples (Ersoy et al., 2021), and based on the observation that in the current sample, the correlation between 14-month IBQ-R fear and 24-month ECBQ shyness (*r* = 0.52 and *p* < 0.001) was greater than the correlation with 24-month ECBQ fear (*r* = 0.46 and *p* < 0.001). In the current sample, internal consistency was good for the fear/shyness (*α* = 0.85–0.89) and the perceptual sensitivity subscales (*α* = 0.85–0.86).

The Social Responsiveness Scale–2 (SRS-2; Constantino & Gruber, 2012) questionnaire designed to measure autistic traits consists of 65 items, each rated on a 4-point scale ranging from 1 (not true) to 4 (almost always true). Parents completed the Preschool forms of the SRS at 36 months and scores on the RRB (12 items) and Social Communication and Interaction subscales (indexing SCI; 53 items) subscales were calculated (henceforth referred to as RRB and SCI). In our sample, both subscales of RRB and SCI showed excellent internal consistency (*α* = 0.99 each). Due to skew, the RRB and SCI variables were log transformed.

### Data analysis

**Figure 1.**
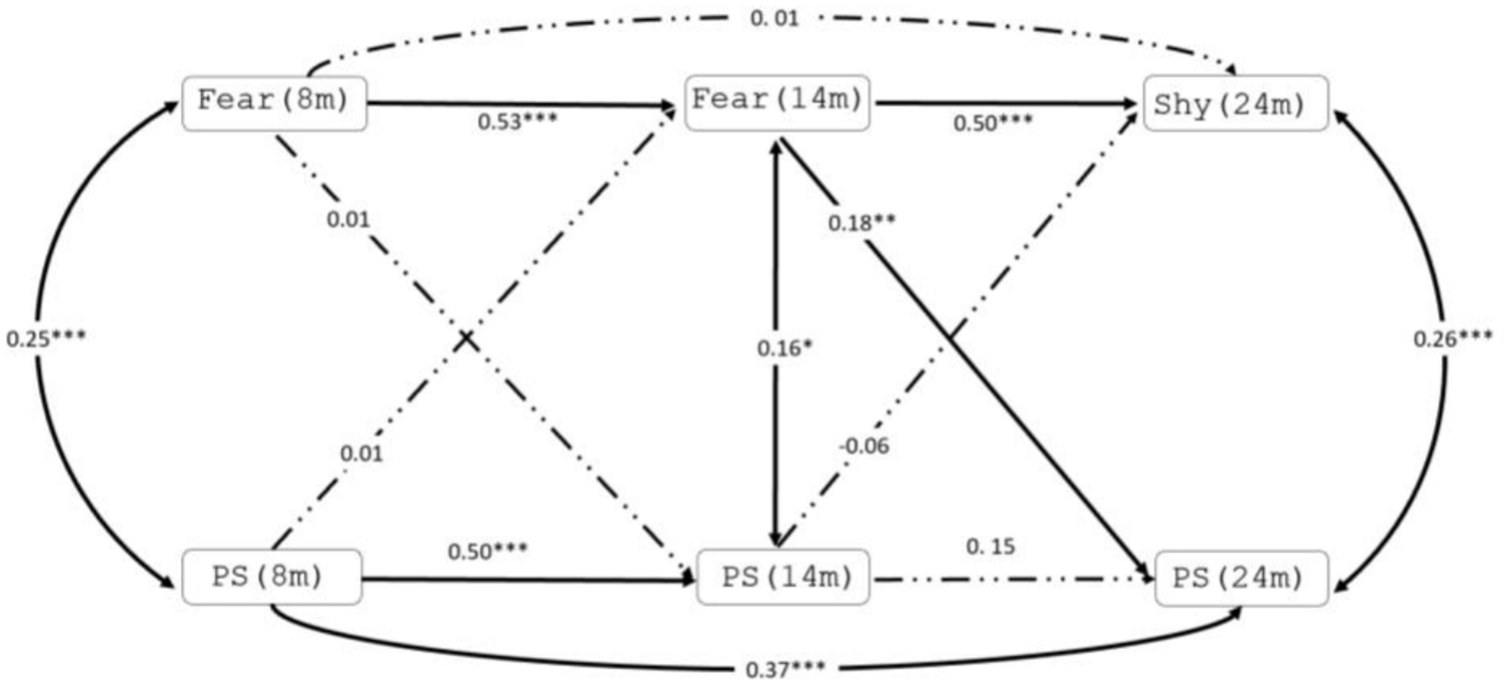
Estimated model for cross-lagged path related to fear/shyness and perceptual sensitivity (PS). Bold indicates significant association. *p < 0.05; **p < 0.01; ***p < 0.001.

**Figure 2.**
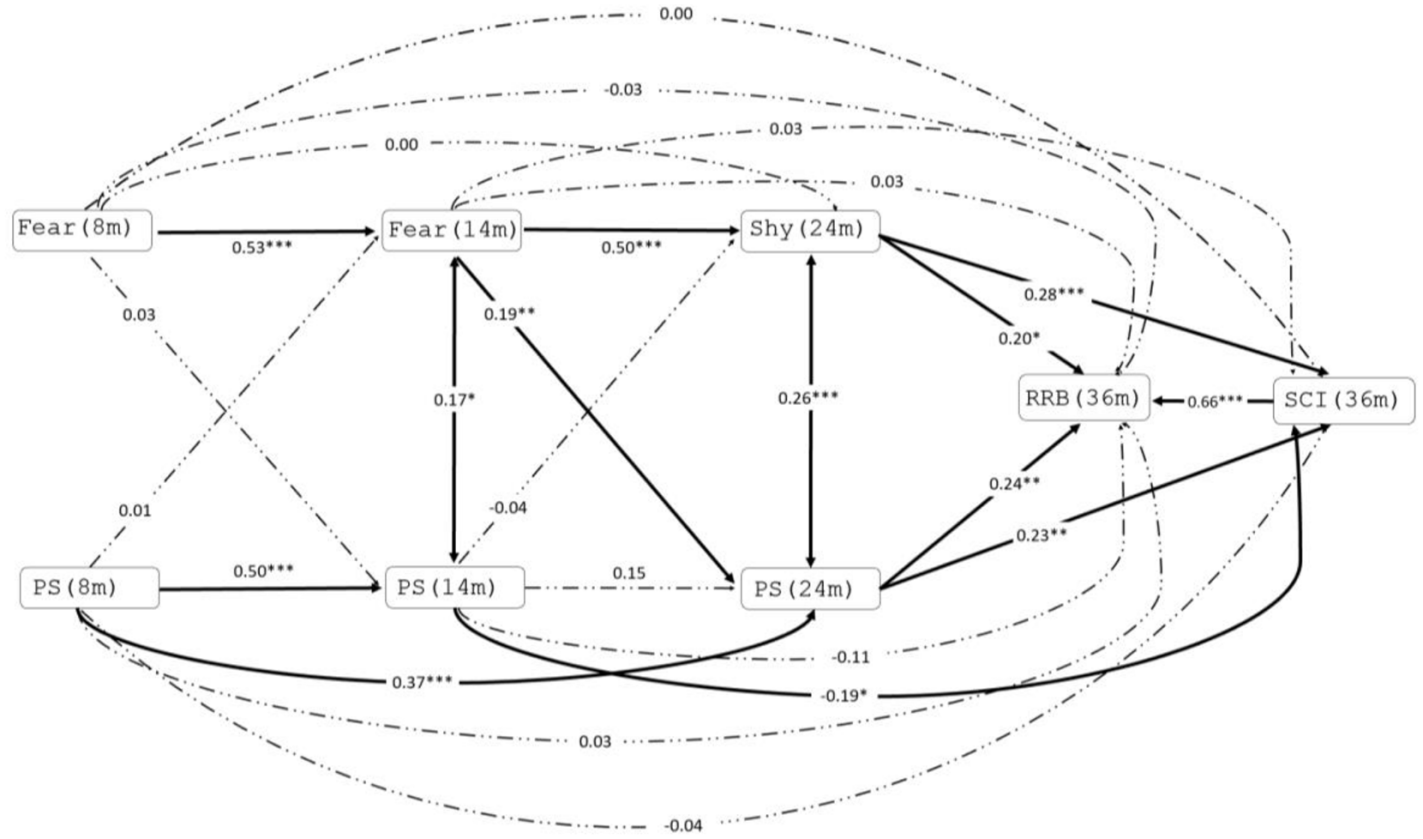
Cross-lagged associations between fear/shyness, perceptual sensitivity (PS), restricted and repetitive behaviours (RRB) and social communication interactions (SCI) 8, 14, 24 and 36 months. Bold indicates significant association. *p < 0.05; **p < 0.01; ***p < 0.001.

Two cross-lagged structural equation models were estimated to test the directionality of pathways between fear/shyness, perceptual sensitivity, RRB and SCI. Model 1 examined the autoregressive and cross-lagged pathways between the three measures of fear/shyness and perceptual sensitivity at 8, 14 and 24 months (Figure 1). Model 2 examined the direction of longitudinal relationships between each timepoint of fear/shyness, perceptual sensitivity measurement and later RRB and SCI (Figure 2). We included SCI as an outcome in Model 2 to assess the specificity of associations to RRB scores; post hoc tests compared the magnitude of the association for any significant predictors of RRB to the magnitude of the association for SCI. All models were estimated using maximum likelihood to account for missing data. For Model 2, robust standard errors were used to correct for any residual skew in RRB/SCI scores. In both models, EL/TL status was adjusted for by entering likelihood status as predictor of all variables in the model. To check that results were not unduly influenced by the subgroup of infants who received an autism diagnosis later in development, we re-ran models excluding these participants (the pattern of findings was largely similar; however, some associations were no longer statistically significant, see Supplementary Table S3 and Figures S1 and S2). Model fit was assessed by the root means square error of approximation (RMSEA) and comparative fit index (CFI). Acceptable fit is indicated by RMSEA of 0.05–0.08 and CFI of 0.90–0.95, whereas good fit is indicated by RMSEA of 0.01–0.05 and CFI of 0.95–1.00 (Hu & Bentler, 1999; Kline, 2016). Model fit indices are not available for models estimated with robust standard errors. We fit models sequentially for parsimony and clarity of interpretation. All models were estimated using observed (i.e. non-latent) variables in STATA 16. For completeness, we also present unadjusted correlation coefficients between fear/shyness and perceptual sensitivity at 8, 14 and 24 months and RRB and SCI at 36 months (see Supplementary Table S2).

### Community involvement

There was no specific community input from autistic individual or family members on the analysis presented in this study. However, the BASIS network views families as partners in our research programme. We regularly hold meetings with a Parent Consultation Group to discuss targeted ethical, procedural and strategic issues at all stages of our work. In 2019, we held a family ‘expo’ event including parents (some of whom had an autism diagnosis), older siblings with an autism diagnosis, and some of the infant siblings who were in mid-childhood, both to share our findings and to gain feedback on our proposed new studies. As part of our work in AIMS-2-TRIALS, we lead regular online meetings of the Ethics and Biomarkers Working Groups in which autistic people help us shape the directions of our research. Understanding co-occurring conditions like anxiety is often brought up by these teams as an important priority, consistent with the results of broader surveys of the community.

## Results

### Sample characteristics

Sample characteristics for all measures and likelihood group comparisons are shown in Table 1. The EL group had higher ADOS social affect scores at 24 months, higher ADOS RRB scores at 24 and 36 months and higher ADI-R scores at 36 months, as compared to the TL group. The TL group had higher scores on MSEL at 8, 24 and 36 months.

**Table 1.** Sample characteristics by Typical and Elevated likelihood groups.

	EL group	TL group	Group difference
8 months			
Sex (*N* girls:*N* boys)	84:85	42:35	*χ*^2^(1) = 0.496, *p* = 0.481
Age in months	8.23 (1.14)	7.91 (1.34)	*t*(244) = −1.941, *p* = 0.053, *d* *=* −0.27
MSEL	102.46 (15.79)	107.25 (12.59)	*t*(244) = 2.344, *p* = 0.020, *d* = 0.32
IBQ-R-fear	2.95 (1.15)	2.54 (0.88)	*t*(233) = −2.735, *p* = 0.007, *d* = −0.39
IBQ-R-perceptual sensitivity	3.79 (1.27)	3.81 (1.15)	*t*(186) = 0.109, *p* = 0.913, *d* = 0.02
14 months			
Sex (*N* girls:*N* boys)	82:84	41:34	*χ*^2^(1) = 0.450, *p* = 0.502
Age in months	14.51 (1.32)	14.35 (1.32)	*t*(239) = −0.870, *p* = 0.385, *d* = −0.12
MSEL	95.20 (15.57)	104.81 (15.26)	*t*(244) = −1.941, *p* = 0.053, *d* = 0.62
IBQ-R-fear	3.47 (1.08)	2.99 (0.88)	*t*(225) = −3.3381, *p* *=* 0.001, *d* = −0.48
IBQ-R-perceptual sensitivity	3.74 (1.23)	4.13 (1.04)	*t*(198) = 2.178, *p* = 0.031, *d* = 0.33
24 months			
Sex (*N* girls:*N* boys)	79:79	38:35	*χ*^2^(1) = 0.346, *p* = 0.556
Age in months	25.39 (1.99)	24.21 (0.93)	*t*(229) = −4.822, *p* *<* 0.001, *d* = −0.68
MSEL	99.69 (20.13)	115.55 (14.14)	*t*(221) = 5.913, *p* *<* 0.001, *d* = 0.86
ADOS CSS SA	3.05 (2.05)	1.96 (0.60)	*t*(182) = −2.689, *p* = 0.008, *d* = −0.57
ADOS CSS RRB	4.09 (2.72)	2.54 (2.20)	*t*(182) = −2.773, *p* = 0.006, *d* = −0.59
ECBQ-shyness	3.33 (1.05)	2.90 (0.82)	*t*(206) = −2.997, *p* = 0.003, *d* = −0.44
ECBQ-perceptual sensitivity	3.32 (1.18)	3.34 (1.13)	*t*(206) = 0.104, *p* = 0.917, *d* = 0.15
36 months			
Sex (*N* girls:*N* boys)	80:84	38:35	*χ*^2^(1) = 0.329, *p* = 0.566
Age in months	38.43 (2.21)	38.40 (2.65)	*t*(235) = −0.108, *p* = 0.914, *d* = −0.02
MSEL	103.39 (23.99)	117.04 (15.91)	*t*(234) = 4.440, *p* *<* 0.001, *d* = 0.63
ADOS CSS SA	3.54 (2.63)	3.33 (2.25)	*t*(235) = −0.603, *p* = 0.547, *d* = −0.08
ADOS CSS RRB	4.55 (2.64)	3.71 (2.47)	*t*(235) = −2.296, *p* = 0.023, *d* = −0.32
ADI-R social	3.80 (4.97)	0.96 (1.49)	*t*(186) = −2.836, *p* = 0.005, *d* = −0.61
ADI-R communication	4.07 (4.73)	0.48 (1.05)	*t*(186) = −3.777, *p* *<* 0.001, *d* = −0.81
ADI-R RRB	1.43 (2.40)	0.08 (0.28)	*t*(186) = −3.065, *p* = 0.003, *d* = −0.66
SRS-RRB	5.24 (7.19)	1.58 (2.19)	*t*(223) = −4.220, *p* *<* 0.001, *d* = −0.60
SRS-SCI	35.48 (26.25)	19.81 (9.24)	*t*(223) = −4.921, *p* *<* 0.001, *d* = −0.70

ADI-R: Autism Diagnostic Interview–Revised; ADOS: Autism Diagnostic Observation Schedule; ECBQ: Early Childhood Behavioural Questionnaire; IBQ-R: Infant Behavioural Questionnaire–Revised; MSEL ELC: Mullen Scales of Early Learning Early Learning Composite Standard Score; RRB: Restrictive and Repetitive Behaviours; SA: Social Affect; SCI: Social Communication Interaction; SD: standard deviation; SRS: Social Responsiveness Scale.

### Model 1: associations between fear/shyness and perceptual sensitivity from 8 to 24 months

The cross-lagged model provided a good fit to the data (*χ*^2^(2) = 3.83, *p* = 0.15; CFI = 0.99, RMSEA = 0.06) (Figure 1). There were significant associations between fear at 8 months and fear at 14 months (*β* = 0.53 and *p* < 0.001) and between fear at 14 months and shyness at 24 months (*β* = 0.50 and *p* < 0.001), but not between fear at 8 months and shyness at 24 months (*β* = 0.01 and *p* = 0.93). Similarly, there were significant associations between perceptual sensitivity at 8 and 14 months (*β* = 0.50 and *p* < 0.001) and 8 to 24 months (*β* = 0.37 and *p* < 0.001), and the association between 14 and 24 months (*β* = 0.15 and *p* = 0.06) scores fell just short of significance. Cross-sectional associations between fear and perceptual sensitivity were significant at 8 (*β* = 0.25 and *p* < 0.001), 14 (*β* = 0.16 and *p* *=* 0.02) and 24 (*β* = 0.26 and *p* < 0.001) months. Cross-lagged paths indicated that higher levels of fear at 14 months were associated with higher levels of perceptual sensitivity at 24 months (*β* = 0.18 and *p* = 0.01). All other cross-lag pathways were non-significant (*p*s ⩾ 0.38).

### Model 2: longitudinal association between fear/shyness, perceptual sensitivity and RRB and SCI at 36 months

There was a concurrent positive association between RRB and SCI at 36 months (*β* = 0.66 and *p* < 0.001). Both higher levels of shyness and perceptual sensitivity at 24 months were significantly associated with heightened levels of RRB (*β* = 0.20 and *p* = 0.01; *β* = 0.24 and *p* < 0.01, respectively) and SCI (*β* = 0.28 and *p* < 0.001; *β* = 0.23 and *p* < 0.01, respectively). Pairwise post hoc tests suggested no differences in the strength of these associations (all *p*s > 0.21). Lower levels of perceptual sensitivity at 14 months were related to higher SCI only (*β* = −0.19 and *p* = 0.02).

### Sensitivity analysis

When models were rerun excluding infants who received an autism diagnosis, the patterns mostly remained the same (see Supplementary Materials). The association between perceptual sensitivity at 8 months and SCI at 36 months became significant (*β* = −0.22 and *p* = 0.023), such that lower levels of perceptual sensitivity were associated with higher levels of SCI. The associations between fear at 14 months and perceptual sensitivity at 24 months (*β* = 0.16 and *p* = 0.034), shyness at 24 months and SCI at 36 months (*β* = 0.23 and *p* = 0.001), perceptual sensitivity at 24 months and SCI at 36 months (*β* = 0.30 and *p* < 0.001) and perceptual sensitivity at 24 months and RRBs at 36 months (*β* = 0.29 and *p* = 0.001) remained significant. The associations between shyness at 24 months and RRB at 36 months (*β* = 0.13 and *p* = 0.140) and perceptual sensitivity at 14 months and SCI at 36 months were no longer statistically significant (*β* = −0.10 and *p* = 0.294).

## Discussion

This study investigated the directionality of associations between fear/shyness and perceptual sensitivity in the first 2 years of life and tested whether these two constructs were associated with later manifestations of RRB and SCI in a longitudinal cohort of infants enriched for autism outcomes. Cross-lag models indicated that at each timepoint, levels of fear/shyness and perceptual sensitivity positively predicted within-domain scores at the next time point, and higher levels of fear at 14 months were associated with higher levels of perceptual sensitivity at 24 months (but not vice versa). This suggests it is possible to measure temperamental fear/shyness and sensory sensitivity from the first years of life with some stability, and that the two domains may be interrelated. Results also showed that higher levels of shyness and perceptual sensitivity at 24 months were associated with heightened levels of both RRB and SCI scores at 3 years, indicating that differences in anxiety and sensory processing may not be specific precursors of RRB but are shared with SCI.

### Group differences and continuity in infant manifestations of anxiety and perceptual sensitivity

We found that those from the EL group scored higher on fear/shyness at all three age points as compared to TL group, highlighting that greater fear/shyness is associated with autism likelihood in infancy. With regard to perceptual sensitivity, the EL group scored lower than TL group on perceptual sensitivity at 14 months, with the direction of effect comparable to an earlier study in our cohort (with partly overlapping participants) (Clifford et al., 2013). Models showed significant autoregressive pathways, suggesting a substantial degree of within-domain continuity for both fear/shyness and perceptual sensitivity in the early infant period (aside from 14 to 24 months perceptual sensitivity which showed a non-significant trend). Our findings concur with research reporting significant within-person continuity in these constructs in young neurotypical and older autistic samples (Green et al., 2012; Putnam et al., 2006), which will be of interest to other researchers seeking to study early infant manifestations and developmental trajectories of these domains in typical and atypical populations. However, we highlight there is always a possibility that parents form a stable view of their child and may report consistency even if there is meaningful behaviour change; multi-respondent longitudinal designs are needed to test this hypothesis.

### Longitudinal associations between fear/shyness and perceptual sensitivity

We extend cross-sectional findings (Lane et al., 2012; Lidstone et al., 2014; Neil et al., 2016; Wigham et al., 2015) by testing the directionality of associations between anxiety and sensory sensitivity in early infancy. Our results showed greater fear at 14 months was associated with higher perceptual sensitivity at 24 months and not vice versa. One interpretation of this finding is that infants may be more likely to notice environmental sensory stimuli if they are hypervigilant of their environment, that is, being startled at sudden or loud noises (a core symptom of anxiety). Our findings are in contrast to previous work which found that sensory over-responsivity predicted anxiety 1 year later in autistic toddlers (Green et al., 2012) (and not vice versa). Differences in ages, sample and the measurement of sensory/perceptual processing may have contributed to these opposing sets of results. The mean age upon entry to the study by Green and colleagues was 28 months, and infants were followed up 1 year later, thus capturing a later developmental period (28–40 months) than that covered in this set of our analyses (8–24 months). Given that the early infant period is characterised by developmental change in how incoming information is processed and responded to, it is possible that the nature of the association between sensory processing and fear/anxiety changes over development. In addition, all participants in the study by Green et al. had a diagnosis of autism, whereas the current sample was made up infants at TL and EL of developing autism, and only a small subset went on to receive a diagnosis themselves. Green and colleagues asked parents specifically about their child’s negative emotional response to sensory stimuli (e.g. ‘is bothered by loud noises or bright lights’), which may have included a more affective sensory response, whereas the current questionnaires were tapping infant’s general sensitivity to the environment (e.g. ‘How often during the last week did the baby appear to listen to even very quiet sounds’). Collecting information on both objective measures of sensory processing (e.g. discrimination thresholds) and affective response to different sensory inputs may help to disentangle the role of processing of, as compared to emotional reactivity to, different sensory inputs in elevating risk for anxiety.

### Longitudinal association between fear/shyness, perceptual sensitivity, RRB and SCI

Results also show that higher levels of shyness and perceptual sensitivity at 24 months are associated with higher levels of parent-rated RRB at age 3. The finding of a positive association between infant manifestations of fearfulness and RRB extends previous cross-sectional studies that report positive associations between anxiety and RRB in older autistic individuals (Gotham et al., 2013; Lidstone et al., 2014; Rodgers et al., 2012) by establishing a longitudinal path from infant anxiety and perceptual processing to later RRB at 36 months. One idea put forward is that RRBs are employed as a strategy to regulate arousal levels and decrease anxiety by controlling environmental input (Lidstone et al., 2014), and our findings support this hypothesis. However, we highlight that we did not have a measure of RRB scores before 24 months (where fear/shyness and perceptual sensitivity were measured). Thus, it could also be possible that RRBs precede anxiety (as found in older samples by Baribeau et al., 2020), or that bidirectional associations best characterise them over time.

Similar to fear/shyness, we also found greater perceptual sensitivity at 24 months was positively associated with RRB at 36 months. Similar results are found in other studies on EL (Wolff et al., 2019) and typically developing (Schulz & Stevenson, 2019) populations, where sensory-related behaviours are significantly associated with a wide range of RRB. Similar to the interpretation for the association between fear/shyness and RRB, if a developing infant is especially sensitive to small changes in their sensory environment, they may develop a preference for sameness/rigid pattern of behaviour to regulate incoming novel sensory information, although more precise measurement of sensory sensitivity as compared to sensory reactivity (as in Schulz & Stevenson, 2019) would help to better test this working hypothesis. Alternatively, in keeping with the fact that sensory processing differences are a subdomain of RRB, these may be among the earliest manifestations of RRB-type symptoms. A more parsimonious interpretation of our findings is that we see associations between these two domains because we are measuring the same construct, which is especially pertinent given sensory processing differences are part of the RRB symptom domain (and thus are probed in the SRS-2 RRB items). However, it seems unlikely this could fully account for the observed association as only one SRS-2 RRB item specifically assessed sensory processing differences (‘Shows unusual sensory interest or strange ways of playing with toys’).

We further assessed whether the relations between fear/shyness and perceptual sensitivity with later RRB are specific to this domain. Contrary to this hypothesis, we found a similar pattern of associations existed with SCI, that is, higher levels of shyness and perceptual sensitivity at 24 months are also associated with higher levels of SCI at 36 months (in line with Vlaeminck et al., 2020), and the pathways from fear/shyness and perceptual sensitivity to RRB and SCI were of comparable strength. This raises the question of specificity. Constructs such as anxiety and sensory processing have been proposed to specifically relate to RRB, but our results suggest they may also be important in understanding the emergence of SCI. These results challenge research that argues for fractionation of the different domains of autistic symptoms, in that they should be conceptualised as independent constructs with differential genetic and cognitive correlates (Happé & Ronald, 2008). Instead, our results suggest that there may be shared developmental pathways to RRB and SCI in toddlerhood (Constantino et al., 2021). It may be that the fractionation of domains of autistic symptoms is developmentally specific in that autistic symptoms manifest as one latent construct early in infancy, but the two domains become more differentiated as individuals become older (although see Beuker et al., 2013). We also highlight that although this study focused on autistic traits as the outcome of interest; and it might be case that infant fear/shyness and perceptual sensitivity are associated with traits/characteristics beyond those indexing autism, such as emotional and behavioural difficulties. This requires investigation in future studies.

Finally, we found *lower* perceptual sensitivity at 14 months was associated with more persistent SCI (but not RRB). As the focus of this article was on infant precursors of RRB, we did not form specific predictions as to the nature of associations with SCI; this domain of autistic characteristics was only included to assess the specificity of associations to RRB. Therefore, we do not consider this result a confirmation of a specific hypothesis, but rather something to be explored further with relevant measures. However, we do note that our results concur with two other longitudinal infant sibling studies, where decreased sensory sensitivity is associated with decreased neural response to social stimuli and fewer social approach behaviours (Jones et al., 2018), and increased sensory seeking (indicative of hypo-sensitivity and/or reduced response to sensory input) is associated with greater social difficulties in toddlerhood through the mechanism of reduced social orienting (Baranek et al., 2018). As many of the items in the perceptual sensitivity measure used at 14 months ask about environmental awareness/noticing, one hypothesis is that if you are less sensitive to incoming sensory information (including social cues) at a critical developmental period, this could lead to atypical development of social cognition and consequent difficulties in social interactions (Jones et al., 2018). These results are interpreted as highlighting the importance of awareness of environmental changes for learning about social information (e.g. through social orienting) and adaptive social development. It may be that there are sensitive periods in early development where it is important for the developing infant to be acutely aware of changes in their ongoing environment to promote social learning whereas, conversely, the same level of sensitivity later in development impedes ongoing social interactions (e.g. as the infant is distracted from social situations by sensory hypersensitivities). Better understanding of the normative developmental trajectories of sensory sensitivity and reactivity in infancy is necessary to delineate how atypical sensory development impacts emerging socio-cognitive abilities.

Finally, we highlight that in sensitivity analyses excluding infants who went onto be identified as autistic, most associations remained (aside from the associations between shyness at 24 months and RRB at 36 months, and perceptual sensitivity at 14 months and SCI at 36 months). The fact the pattern of findings largely remained (and even those that became non-significant had comparable coefficients of effect) suggests that the reported associations may represent mechanisms that are present across typical and atypical development, in line with studies suggesting the comparability of genetic influences on binary diagnostic status versus continuous variation in traits (Robinson et al., 2011).

### Strengths and limitations

The main strengths of this study are the prospective longitudinal design, where infants are followed from birth to 36 months of age, giving a detailed picture of dynamic developmental changes in the first few years of life, and the moderate-to-large sample size for this type of study. This longitudinal approach is key to examining the directionality of early emerging associations in a reasonably large sample. However, we also note some limitations. As we solely used parent-report measures, shared method variance may have contributed to associations between the domains. In addition, the fact that in some families, there was already a first-degree relative with autism may have impacted how parents report on the behaviour of infant siblings. For example, parental perception of the older sibling with autism may influence their reporting on younger siblings’ behavioural traits. Nevertheless, in previous study, parent report and direct observation ratings overlapped moderately for sensory over-responsivity (Tavassoli et al., 2019). It is also not well-known whether scores on the IBQ are influenced by other autistic characteristics (e.g. social motivation and monotropism). Furthermore, we highlight a change in the questionnaire from IBQ-R at 14 months to its counterpart at 24 months (ECBQ) to ensure that items are age-appropriate. Despite this change in questionnaires, we found substantial within-domain continuity in fear/shyness and perceptual sensitivity over time, suggesting that the change of instrument did not have an overly large impact on construct measurement. Future research should focus on incorporating experimental and observational measures of infant anxiety (such as changes in heart rate and skin conductance), sensory processing (including brain measures to distinguish reactivity from detection and habituation) and autistic symptoms (e.g. observation and video coding methods; Damiano et al., 2013; Harrop et al., 2014) to minimise the impact of shared method variance and measurement overlap and disentangle sensory sensitivity as compared to sensory responsiveness and affective reactivity. In addition, measures of anxiety and perceptual sensitivity that can be used across a wide developmental range (e.g. auditory oddball paradigms paired with psychophysiological recording; Haartsen et al., 2019) may be a useful source of complementary information to age-dependent questionnaires. Finally, although we have conceptualised fear/shyness and perceptual sensitivity as separable individual characteristics that are associated with later autism traits, it is unclear whether they represent constructs which influence the expression of autism traits later in development (e.g. the proposed hypothesis that being less sensitive to incoming sensory information impacts the development of social cognition), or are simply early markers of emerging autism (Constantino et al., 2021; Johnson et al., 2021).

## Conclusion

This study found early infant manifestations of anxiety (e.g. fear/shyness) were associated with later perceptual sensitivity, and higher fear/shyness and perceptual sensitivity at 24 months were both associated with more RRB and SCI measured 1 year later. We also found lower perceptual sensitivity at 14 months was associated with greater SCI scores in toddlerhood. Findings build on cross-sectional work on the correlates of RRB, but also call into question theories that argue that these domains specifically underlie RRB rather than the broader range of autistic characteristics. Given the possible cascading effects of early anxiety and sensory atypicalities on autism symptoms, our findings support the importance of further research to increase our understanding of those children who are likely to develop autism, and to guide future attempts to develop mechanistically informed early intervention and supports.

## Supplemental Material

sj-docx-1-aut-10.1177_13623613211068932 – Supplemental material for A prospective study of associations between early fearfulness and perceptual sensitivity and later restricted and repetitive behaviours in infants with typical and elevated likelihood of autism Supplemental material, sj-docx-1-aut-10.1177_13623613211068932 for A prospective study of associations between early fearfulness and perceptual sensitivity and later restricted and repetitive behaviours in infants with typical and elevated likelihood of autism by Nisha Narvekar, Virginia Carter Leno, Greg Pasco, Mark H Johnson, Emily JH Jones and Tony Charman in Autism
