# Supplementary material for: A prospective study of associations between early fearfulness and perceptual sensitivity and later restricted and repetitive behaviours in infants with typical and elevated likelihood of autism
Source: Autism. 2022 Jan 13;26(8):1947–58. doi: 10.1177/13623613211068932 (PMC9597143; doi:10.1177/13623613211068932)
Supplement: sj-docx-1-aut-10.1177_13623613211068932 – Supplemental material for A prospective study of associations between early fearfulness and perceptual sensitivity and later restricted and repetitive behaviours in infants with typical and elevated likelihood of autism [file sj-docx-1-aut-10.1177_13623613211068932.docx]

**Supplements:**

**Table S1** Sample characterization by phase and outcome groups.

|  | EL Autism | EL Atypical | EL Typical | TL |
| --- | --- | --- | --- | --- |
| Phase 1 |  |  |  |  |
| Sex (N girls: N boys) | 6:11 | 9:3 | 17:7 | 29:21 |
| Phase 2 |  |  |  |  |
| Sex (N girls: N boys) | 2:15 | 12:20 | 36:28 | 13:14 |
|  |  |  |  |  |

**Table S2** Whole Sample Correlations between Fear/Shyness, Perceptual Sensitivity, RRB and SCI.

IBQ Fear IBQ Fear ECBQ Shyness IBQ PS IBQ PS ECBQ PS SRS RRB SRS SCI

8 months 14 months 24 months 8 months 14 months 24 months 36 months 36 months

----------------------------------------------------------------------------------------------------------------------------------------------------------------------------------------------------------------

IBQ-R Fear 8m 1.000 (235)

IBQ-R Fear 14m 0.563* (219) 1.000 (227)

ECBQ Shy 24m 0.300* (202) 0.515* (196) 1.000 (208)

IBQ-R PS 8m 0.251 (186) 0.144 (178) 0.088 (159) 1.000 (188)

IBQ-R PS 14m 0.105 (194) 0.179 (195) 0.042 (174) 0.514* (163) 1.000 (200)

ECBQ PS 24m 0.292* (201) 0.252 (195) 0.306* (203) 0.488* (161) 0.357* (173) 1.000 (208)

SRS RRB 36m 0.159 (213) 0.219 (208) 0.326* (197) 0.141 (172) -0.015 (186) 0.254* (197) 1.000 (225)

SRS SCI 36m 0.181 (213) 0.256*(208) 0.411* (197) 0.018 (172) -0.112 (186) 0.203 (195) 0.729* (225) 1.000 (225)

() sample size

* Correlation is significant at the p<.01 level

ECBQ = Early Childhood Behavioral Questionnaire; IBQ-R = Infant Behavioral Questionnaire-Revised; PS = Perceptual Sensitivity; RRB = Restrictive and Repetitive Behaviors; SCI = Social Communication Interaction; SRS = Social Responsiveness Scale.

**Table S3** Summary of selected path analysis results. Standardised beta coefficient and their p-values

|  | Standardised beta coefficient, p-values |
| --- | --- |
|  |  |
| Fear 8m – Fear 14m | β = 0.52, *p <* .001 |
| Fear 14m – Shyness 24m | β = 0.47, *p <* .001 |
| Shyness 24m – SCI 36m | β = 0.23, *p =* .001 |
| Perceptual Sensitivity 8m - Perceptual Sensitivity 14m | β = 0.52, *p <* .001 |
| Perceptual Sensitivity 8m - Perceptual Sensitivity 24m | β = 0.39, *p <* .001 |
| Perceptual Sensitivity 24m – SCI 36m | β = 0.30, *p <* .001 |
| Perceptual Sensitivity 8m – SCI 36m | β = -0.22, *p =*.023 |
| Fear 14m – Perceptual Sensitivity 24m | β = 0.16, *p =* .034 |

SCI = Social Communication Interaction


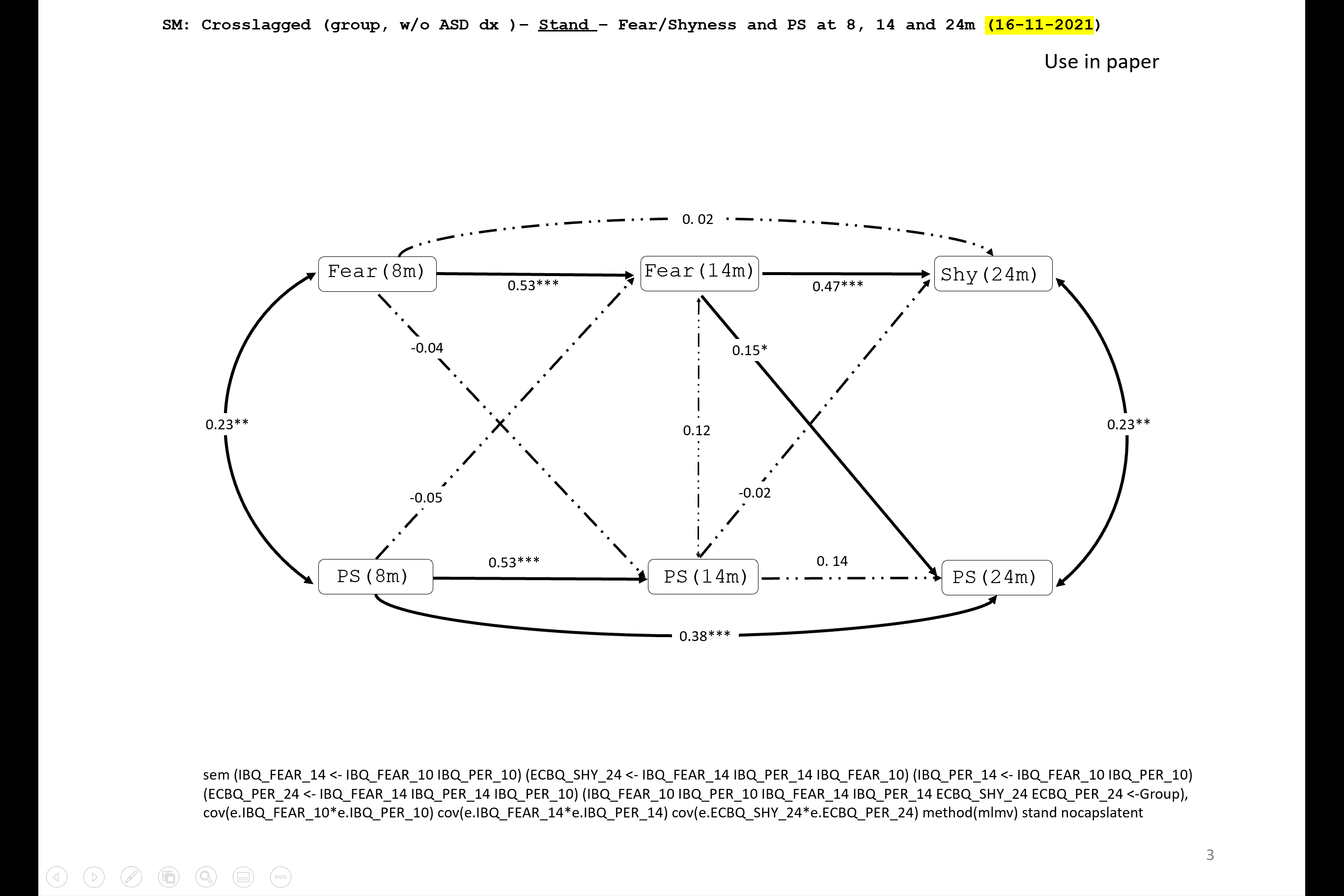


**Fig. S1** Estimated model for cross lagged path related to Fear/Shyness and Perceptual Sensitivity (PS) in infants without autism outcomes. Bold indicates significant association. (**p* < .05, ***p* < .01, and *** *p* < .001)


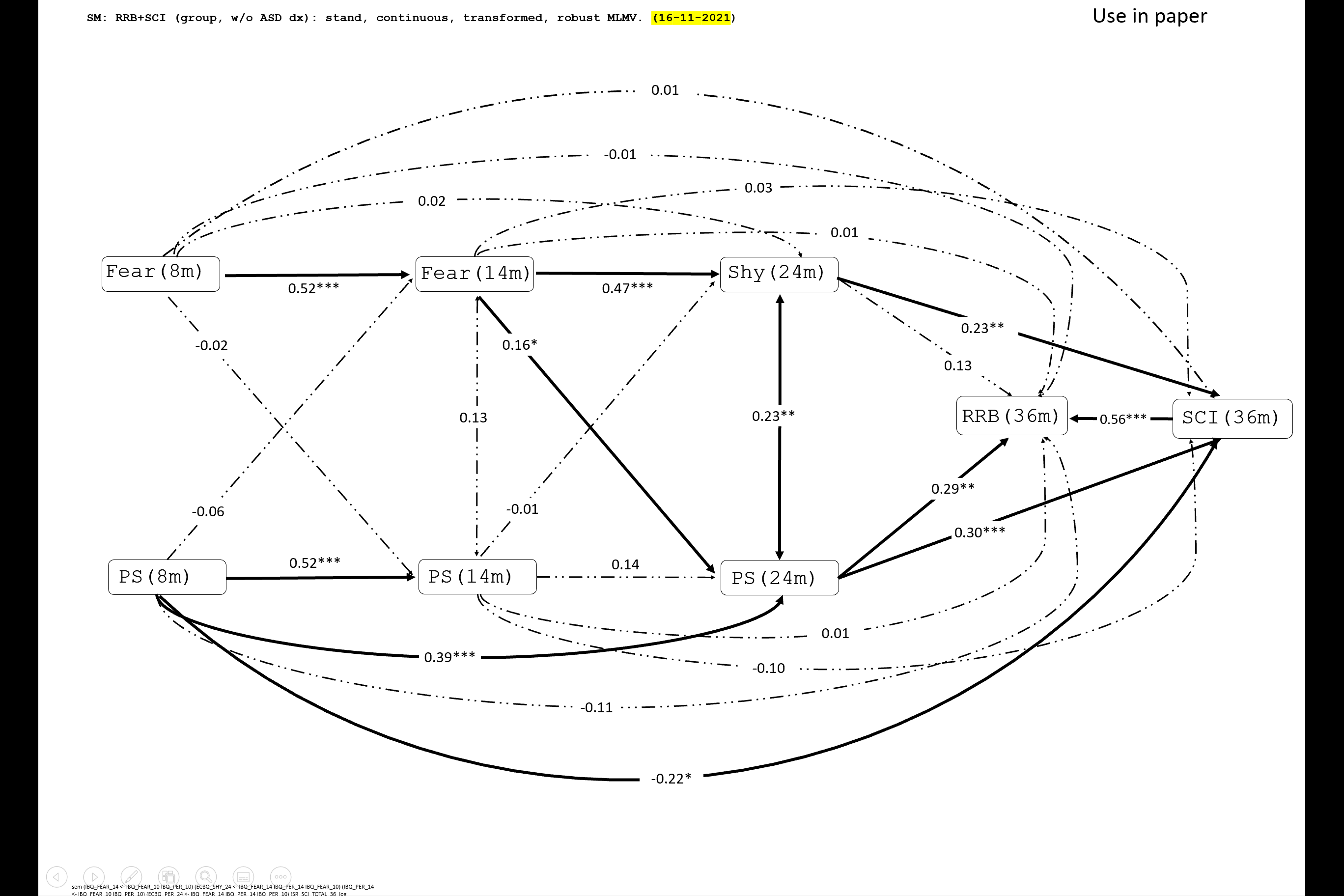


**Fig. S2** Cross-lagged associations between Fear/Shyness, Perceptual Sensitivity (PS), restricted and repetitive behaviours (RRB) and social communication interaction (SCI) 8, 14, 24 and 36 months in infants without autism outcomes. Bold indicates significant association. (**p* < .05, ***p* < .01, and *** *p* < .001).
